# Germany’s education policy during the COVID-19 crisis

**DOI:** 10.1007/s41358-021-00262-7

**Published:** 2021-05-31

**Authors:** Vera Freundl, Philipp Lergetporer, Larissa Zierow

**Affiliations:** ifo Institute – Leibniz Institute for Economic Research at the University of Munich e. V., Poschingerstr. 5, 81679 Munich, Germany

## Introduction

The COVID-19 pandemic is having a massive impact on almost all areas of life, and poses unprecedented challenges for many policy makers around the globe. Given the dynamics of the pandemic, politicians are forced to take decisions that bear direct repercussions on citizens’ health, economic situation, and personal liberties on a daily basis. Naturally, these decisions are under close public scrutiny, and many yield major controversies in the public discourse. The fact that several important elections take place in Germany in this year—including the federal election and six state elections—raises the question of how pandemic-related policy making may affect electoral results. In this article, we argue that education policy may be one of the key policy areas that determine citizens’ voting behavior.

To inhibit the spread of the COVID-19 pandemic, a large share of schools around the globe were closed for in-person teaching for several months (e.g., UNESCO 2021a). These schooling disruptions yielded substantial learning losses around the world (see, for instance, Engzell et al. (2021); Maldonado and de Witte (2020), and Chetty et al. (2020), for evidence from the Netherlands, Belgium and the U.S., respectively). In Germany, the learning time of school children was reduced dramatically as a consequence of the school closures: During the first COVID-19-related school closures in spring 2020, they only spent an average of 3.6 h per day on learning activities, compared 7.4 h before the closures (Grewenig et al. 2020). Education policy did not succeed in compensating these large learning losses, since teachers and schools only carried out a fraction of their usual teaching operations during the school closures. Disappointingly, more than one year after the onset of the COVID-19 pandemic, German education policy still lacks consistent rules on when to close schools, binding distance-teaching concepts, and a viable path how to organize remote tests and final examinations.

We argue that the inability of education policy makers in Germany to ensure adequate education for all children during the COVID-19 pandemic may influence voting decisions in this year’s elections. There is mounting evidence that large parts of the German electorate consider education a policy area that is important for their voting behavior (Woessmann et al. 2017). Furthermore, recent research established a strong link between school children’s educational performance and the electorate’s satisfaction with their state’s education policy making (Woessmann et al. 2020). While legislative and executive power over education policy is under the responsibility of the German states, the federal government intervened in education policy on several occasions during the pandemic. Thus, education policy may not only play a role in state elections, but also in the federal election. Furthermore, education policies—and children’s learning losses induced by school closures—were featured prominently in public debates since the start of the pandemic. Therefore, voters may not be willing to re-elect the party that currently provides the respective education minister who failed to counteract COVID-19-induced learning losses in the past year. Finally, the increased salience of education policy in the public discourse likely renders education policy even more important for voting decisions than it was before the pandemic.

The remainder of the paper is structured as follows. Sect. 2 provides some institutional background on schooling during the COVID-19 pandemic in Germany. Sect. 3 discusses evidence on learning losses due to the COVID-19-related school closures. Sect. 4 presents several reasons as to why education policy may impact voting decisions in this years’ elections. Sect. 5 concludes.

## Institutional background: German education policy during the pandemic

Since the onset of the COVID-19 pandemic in early 2020, the novel virus has caused severe disruptions to everyday life in countries around the world. Ever since, governments have taken various measures to reduce social contacts in an attempt to contain the spread of the virus. A widely adopted course of action was the closure of schools and other educational institutions. Indeed, in the first half of 2020, over 90% of school children worldwide (1.5 billion) were hit by fully or partially closed schools. One year into the pandemic, school closures still affect almost 50% of the world’s students (UNESCO 2021a). This is also true for Germany: In April 2021, schools of an estimated 10 million children had to be (partially) closed because of the pandemic (UNESCO 2021b).

In Germany, the spread of COVID-19 commenced in late January 2020 with the reporting of the first official case. Soon, Germany faced quickly rising infection numbers, and both federal and local governments enforced contact limitations and other social-distancing requirements. School closures were first introduced by one district on February 28 after having recorded a local spike in infection numbers. Two weeks later—on March 13—educational facilities were shut down nationwide (Anger et al. 2020). This decision was taken by the 16 federal states as these hold the legislative and executive power over public education. The only exemption to this measure were young children of parents in so-called system-relevant occupations (e.g., health, public safety, public transportation, and groceries) who could attend emergency services in schools (*Notbetreuung*).

This first period of school closures in Germany was characterized by a lack of centralized concepts for distance teaching and remote learning, leaving the implementation of such measures at the discretion of the individual schools and teachers.^1^ In the second half of April 2020, the state education ministers decided to gradually re-open schools.^2^ Students in graduation classes were the first ones to return to school. During May and June, also other grades re-started in-person teaching, often on a reduced schedule with alternating halves of students per classroom in daily or weekly shifts, accompanied by strict hygiene rules. Again, there was no national strategy on school openings, and the specific timing and the procedures implemented varied widely across federal states.

After the summer break, schools re-opened in September—initially, with in-person teaching for all students. State education ministers proclaimed that everything should be done to ensure in-person teaching, and that open schools should be a priority over other societal concerns.^3^ However, the second wave of rising COVID-19 infection numbers in Germany worsened the situation during the months of October and November so that the chancellor Angela Merkel and the heads of the federal states jointly decided for nation-wide school closures in mid-December. Schools remained almost completely closed for in-person teaching for about two months for most students, and gradually re-opened based on state-specific guidelines thereafter.

An interesting feature of Germany’s education policy during the COVID-19 pandemic is that the lines of responsibility for education decision making seemed to become more and more blurred. While by constitution, the federal states are generally responsible for public education, a number of recent education-policy decisions have been jointly taken by the state ministers and chancellor Angela Merkel—for instance, the decision to close schools at the end of 2020.^4^ Additionally, to compensate for the loss of learning time caused by the school closures, the Federal Ministry of Education is currently planning a comprehensive tutoring program at a total cost of around 1 billion euros.^5^ These federal decisions were prominently featured in the media and the public discourse at large.

## The impacts of the pandemic on school children

In general, all existing studies on the impacts of school closures find substantial detrimental consequences on students’ learning. For instance, in the Netherlands, the eight-week school closures in spring 2020 resulted in a loss of students’ performance in achievement tests as large as 20% (Engzell et al. 2021). Unfortunately, since no comparable dataset is available for Germany, the effects of the school closures on students’ skills and knowledge are mostly unknown.

In contrast to actual skills, learning-time losses due to the COVID-19-induced school closures in Germany are well-documented. In particular, Grewenig et al. (2020) draw on a large-scale online survey of parents of school children conducted after the first period of school closures in June 2020. The authors collected detailed time-use data on how many hours students spent with a range of different activities per day both before and during the period when schools were closed. The amount of time students spent on school-related activities was cut in half during the school closures, from 7.4 h to 3.6 h per day. More than one-third of students only spent a maximum of 2 h per day on school-related activities, and three-quarters spent at most 4 h.

To substitute the reduced learning time, children substantially increased time spent on more detrimental activities such as watching TV, playing computer games, or consuming social media (from 4.0 h to 5.2 h per day). In contrast, time spent on more conducive activities such as reading, playing music, creative work, or physical exercise increased only slightly from 2.9 h to 3.2 h. In sum, these results suggest that the school closures led to substantial skill and knowledge losses among students in Germany. Furthermore, in their heterogeneity analysis, Grewenig et al. (2020) find that lower-performing students decreased their learning time significantly more than higher-performing students, implying that the school closures further aggravated educational inequality.

Turning to education policy, Grewenig et al. (2020) also study the extent to which schools and teachers succeeded in mitigating the detrimental impact of the school closures through distance-teaching activities. Disappointingly, schools and teachers only carried out a fraction of their usual teaching operations during the school closures. For example, only 29% of students had online classes more than once a week, and only 6% had them daily. Students had even less individual contact with their teachers: Only 17% had contact more than once a week. For almost all children and adolescents, the main teaching mode during the school closures was to complete provided exercise sheets. 87% received exercise sheets more than once a week, but only 37% received feedback on the completed exercises more than once a week. In sum, education policy during this period was largely unsuccessful in compensating the losses in learning time induced by the school closures. Unsurprisingly, more than a third of surveyed parents state that the school closures were a great psychological burden for their child and for themselves.

The unpredictable nature of the first COVID-19-related school closures in spring may justify the inability of education policy to compensate for the loss of in-person instructional time with appropriate distance-teaching concepts. In contrast, the second school closures at the beginning of 2021 were foreseeable and policy makers had sufficient lead time. Yet, they have still not established distance-learning concepts that would ensure appropriate schooling when schools are closed. In their parent survey on student learning during the second school closures, Woessmann et al. (2021) show that learning time was only slightly higher than during the first school closures, but nowhere near the level prior to the COVID-19 pandemic. In addition, the majority of parents report that the effectiveness of learning at home is lower compared to learning at school. Thus, ignoring urgent appeals from parents and scientists, the responsible actors have failed to implement policies to prevent further COVID-19-related learning losses.

In view of the probability of yet another period of closed schools later this year, it remains to be feared that the learning losses incurred will not only not be compensated for, but increase even further. These learning losses have serious economic consequences for both the affected students’ future earnings perspectives and the prosperity of society at large (e.g., Woessmann 2020). By the end of the century, the costs of the school closures could amount to trillions. In Germany, this calculation was highly discussed in the media.^6^

## The importance of education policy for voting behavior

More than one year after the first COVID-19-induced school closures, education policy makers in Germany still have not succeeded in establishing distance learning concepts that ensure appropriate education for all children. In this section, we argue that these failures in education policy making may well affect voting behavior in this year’s federal and state elections.

At the most basic level, a consistent finding from opinion surveys in Germany is that many consider education a policy area that is important for their vote choice. For instance, the ifo Education Survey, an annual representative opinion survey on education policy conducted in Germany since 2014, shows that about two thirds of Germans say that education policy is “very” or “somewhat” important for their vote choice in state elections (e.g., Woessmann et al. 2017).^7^ Furthermore, 76% of respondents find it important that Germany performs well on the PISA test compared to other countries, suggesting that high student performance is a key dimension along which German voters evaluate their education ministers.

Consistently, Woessmann et al. (2020) establish a strong link between student performance and the population’s satisfaction with state education policy: They asked a representative sample of Germans how satisfied they are with the education policy in their respective federal state. This satisfaction measure is strongly related to math performance of 9th graders in a comparative student assessment study (“IQB Bildungstrends 2018”): While more than half (58%) of respondents in states with above-average student performance are “very” or “somewhat” satisfied with their states’ education policy, only 43% and 40% are satisfied in states with mean or below-mean performance, respectively.^8^ This gap in policy satisfaction further widens when respondents are informed about the level of student performance across states: Among the randomly chosen subgroup of respondents who are provided with ranking information, 82% in above-mean performing states are satisfied with their states’ education policy, whereas this is the case for only 24% in states with below-mean performance.

The existing evidence that education policy in general—and education performance in particular—is important for German citizen’s vote choice and satisfaction with education policy carries several implications for how education policy during the COVID-19-pandemic may affect voting behavior in this year’s elections. First, while legislative and executive power over education policy is vested in the German states, the federal government intervened into education policies on several occasions during the pandemic (see Sect. 2 for details). Thus, voters may perceive the federal government as responsible for education decisions, implying that education policy may not only play a role in state elections, but also in this year’s federal election. Second, the finding that voters focus on educational performance when assessing education policy may lend itself to the following directional prediction: Since education performance in Germany decreased dramatically during the pandemic, voters may be reluctant to re-elect the party that currently provides the respective education minister who failed to counteract COVID-19-induced learning losses in the past year. Third, and related, education policy has been under exceptional public scrutiny since the beginning of the pandemic, not least because decisions to close schools have direct consequences not only for the children themselves, but also for their families. This increased salience in the public discourse suggests that education policy will be even more important for voting decisions than it was before the pandemic.

## Concluding remarks

More than one year after the first COVID-19-induced school closures, and despite urgent appeals from parents and scientists, education policy makers in Germany still have not succeeded in establishing distance learning concepts that ensure appropriate education for all children. In this article, we have argued that education policy, and especially the mistakes made over the past pandemic year may affect voter decisions in this year’s elections in Germany. While the outcomes of the elections do, of course, not only depend on education policy, but on a much broader set of factors, one may hope that this year’s elections provide education policy makers with incentives to finally implement adequate distance teaching concepts in precaution for future school closures, and to set up intensive tutoring programs to mitigate the learning losses that have already materialized.

## Footnotes

1 See Kultusministerkonferenz, 13 March 2020, https://www.kmk.org/presse/pressearchiv/mitteilung/detail/News/zum-umgang-mit-dem-corona-virus.html [accessed 15 April 2021].

2 See Kultusministerkonferenz, 16 April 2020, https://www.kmk.org/aktuelles/artikelansicht/hubig-kmk-bereitet-schuloeffnungen-vor.html [accessed 15 April 2021], and Kultusministerkonferenz, 28 April 2020, https://www.kmk.org/dokumentation-statistik/beschluesse-und-veroeffentlichungen/bildung-schule/allgemeine-bildung.html#c7795 [accessed 15 April 2021].

3 See Kultusministerkonferenz, 10 October 2020, https://www.kmk.org/presse/pressearchiv/mitteilung/detail/News/kmk-schulen-muessen-offen-bleiben-gesundheits-und-infektionsschutz-hat-oberste-prioritaet.html [accessed 15 April 15 2021]

4 See Bundesregierung, 13 December 2020, https://www.bundesregierung.de/breg-de/aktuelles/telefonkonferenz-der-bundeskanzlerin-mit-den-regierungschefinnen-und-regierungschefs-der-laender-am-13-dezember-2020-1827392.pdf [accessed 15 April 2021], and Bundesregierung, 10 February 2021, https://www.bundesregierung.de/breg-de/suche/regierungspressekonferenz-vom-10-februar-2021-1852774 [accessed 15 April 2021].

5 See Deutschlandfunk, 27 March 2021, https://www.deutschlandfunk.de/bildung-karliczek-plant-bundesweites-corona.1939.de.html?drn:news_id=1242143 [accessed 15 April 2021].

6 Calculations according to which the societal costs of school closures could amount to trillions of euros by the end of the century have been featured prominently in the German discourse (see, for instance, Der Spiegel, 8 February 2021, https://www.spiegel.de/wirtschaft/soziales/corona-lockdown-der-schulausfall-wird-zur-billionen-bombe-a-80ac06c2-1156-4d79-b894-8266a5bac148 [access 15 April 2021]).

7 Question wording in the ifo Education Survey: “How important is the issue of school and education policy for your personal voting decision in state elections?”. Answer categories: “Very important”, “Somewhat important”, “Somewhat unimportant”, “Not important at all”.

8 States with above-mean mathematics performance in the IQB Bildungstrends 2018 are Saxony, Bavaria, and Thuringia. States with mean performance: Baden-Wuerttemberg, Brandenburg, Saxony-Anhalt and Lower Saxony. States with below-mean performance: Hessen, Rhineland-Palatinate, North Rhine-Westphalia, Hamburg, Schleswig-Holstein, Mecklenburg-Western Pomerania, Saarland, Berlin, and Bremen (see Mahler and Kölm 2019).
